# When barcoding fails: development of diagnostic nuclear markers for the sibling caddisfly species *Sericostoma personatum* (Spence in Kirby & Spence, 1826) and *Sericostoma flavicorne* Schneider, 1845

**DOI:** 10.3897/zookeys.872.34278

**Published:** 2019-08-20

**Authors:** Sonja Darschnik, Florian Leese, Martina Weiss, Hannah Weigand

**Affiliations:** 1 Aquatic Ecosystem Research, Faculty of Biology, University of Duisburg-Essen, Essen, Germany; 2 Centre for Aquatic and Environmental Research (ZWU), University of Duisburg-Essen, Essen, Germany; 3 Musée National d’Histoire Naturelle, Luxembourg, Luxembourg

**Keywords:** Freshwater biodiversity, molecular species identification, RFLP, Sericostomatidae, Trichoptera

## Abstract

The larval stages of the central European sibling caddisfly species *Sericostoma personatum* (Spence in Kirby and Spence, 1826) and *S. flavicorne* Schneider, 1845 are morphologically similar and can only be distinguished by differences in coloration in late larval instars. Identification using the mitochondrial barcoding gene, i.e., the Cytochrome c Oxidase 1, is impossible, as both species share the same highly differentiated haplotypes due to introgression. Nuclear gene markers obtained through double digest restriction site associate sequencing (ddRAD seq), however, can reliably distinguish both species, yet the method is expensive as well as time-consuming and therefore not practicable for species determination. To facilitate accurate species identification without sequencing genome-wide markers, we developed nine diagnostic nuclear RFLP markers based on ddRAD seq data. The markers were successfully tested on geographically distinct populations of the two *Sericostoma* species in western Germany, on known hybrids, and on another sericostomatid caddisfly species, *Oecismus monedula* (Hagen, 1859) that sometimes shares the habitat and can be morphologically confounded with *Sericostoma*. We describe a simple and fast protocol for reliable species identification of *S. personatum* and *S. flavicorne* independent of the life cycle stage of the specimens.

## Introduction

Macroinvertebrate species are important indicators for the ecological status and water quality of freshwater ecosystems. Correct taxa lists are the basis for bioassessments and hence reliable tools for taxonomic identification of species are essential. However, reliable morphological species identification can be difficult or impossible, especially for closely-related species or early larval stages, because diagnostic characters are lacking or not yet visible. To deal with this problem, DNA-based methods have been developed for species identification, i.e., DNA barcoding (Hebert et al. 2003). Here, species identification is based on comparing sequences of standardized marker genes, in the case of macroinvertebrates typically the mitochondrial Cytochrome c Oxidase 1 gene (CO1), with a reference database. However, using only mitochondrial genes can lead to wrong species delimitation when speciation has occurred relatively recently and rapidly (e.g. Schlick-Steiner et al. 2010; Weiss et al. 2018) or when incomplete lineage sorting (Toews and Brelsford 2012), hybridization and introgression (e.g. Weigand et al. 2017), or selection on maternally inherited traits (Toews et al. 2014; Morales et al. 2015) has led to mito-nuclear discordance patterns.

One taxon that is typically considered in freshwater ecosystem monitoring is the caddisfly family Sericostomatidae (Pitsch 1993; Neu 2018). However, morphological identification in this family is problematic for many species because morphological traits can be very similar between, or highly variable within species, resulting in uncertainties concerning the number of species (Malicky 2005). One example are the two central European sibling caddisfly species *Sericostoma personatum* (Spence in Kirby & Spence, 1826) and *S. flavicorne* Schneider, 1845 (Schmidtke and Brandt 1995; Leese and Wagner 2005; Weigand et al. 2017). Both species are morphologically very similar, yet adults can be typically distinguished by sexual organs and by size differences in the maxilliary palps of males (Malicky 2005). Also, whether the name *S. flavicorne* used here should actually be applied only to a species found in Turkey (Sipahiler 2000) and the central European one should be referred to as *S. schneideri* (Botosaneanu, 2001) cannot be solved. Therefore, we stick to the taxonomic assignment proposed by Malicky (2005) calling the species *S. flavicorne*. Larvae might be discriminated at late larval stages based on mesonotum coloration (Weigand et al. 2017; Neu 2018). Furthermore, conflicting patterns between morphological and mitochondrial data concerning species identity were found (Leese and Wagner 2005) and raised the question if morphospecies or mitochondrial lineages represent valid species. To answer that question, Weigand et al. (2017) analyzed genome-wide nuclear single nucleotide polymorphisms (SNPs) for the two *Sericostoma* sister species in Germany obtained by double digest restriction site associated DNA sequencing (ddRAD seq, Peterson et al. 2012). They also compared results to morphometric traits and CO1 sequences. The analysis revealed that there are two distinct nuclear clusters corresponding to the morphospecies, whereas mitochondrial lineages did not match this pattern, as several haplotypes were shared between the nuclear clusters. Historical introgression and repeated ongoing hybridization among the two sibling species were assumed to be the causes for the mito-nuclear discordance pattern. Yet, the distinct nuclear clusters of specimens occurring in sympatry with only occasional F1 and F2 hybrids (Weigand et al. 2017) suggest that both morphospecies represent two distinctly evolving entities.

While the two species are not assessed individually in current biodiversity assessments of streams because of the difficulty to distinguish them, it is known that they differ in their ecological requirements (Pitsch 1993). Hence, a method allowing to discriminate the two species would improve bioassessment, especially when late larval stages and adults are not available. As traditional DNA barcoding fails to distinguish *S. personatum* and *S. flavicorne* and ddRAD seq is too expensive and time-consuming outside scientific research projects, an alternative delimitation method is needed for routine analyses. One fast, reliable and cheap candidate method is the use of diagnostic restriction fragment length polymorphism (RFLP) markers. The method is based on fixed sequence differences between species in palindromic recognition sites for restriction enzymes so that only the DNA of one species contains the recognition site. After detecting these diagnostic differences, corresponding regions are amplified via PCR, digested with chosen restriction enzymes and fragment lengths can be analyzed using agarose gel electrophoresis, thereby omitting sequencing and speeding up species identification. In this study, we developed a set of nine diagnostic RFLP markers using the nuclear ddRAD seq data as well as the draft genome from Weigand et al. (2017). To assess their reliability and performance for the identification of *S. flavicorne*/*personatum* in Germany, the markers were tested in single and multiplex reactions with samples from two different geographic regions. Additionally, the markers were tested for *Oecismus monedula* (Hagen, 1859), which often co-occurs in the stream habitats and can be mis-identified as *Sericostoma*, especially in early larval stages and when coloring is ambiguous.

## Materials and methods

### Identification of diagnostic markers

For identification of diagnostic RFLP markers, the ddRAD seq data from Weigand et al. (2017) were used. As ddRAD data and data from the current study originated only from few geographically restricted regions in Germany and no type material was included, the species affiliation to *S. flavicorne* and *S. personatum* may be erroneous (Botosaneanu 2001, Sipahiler 2000). However, it reflects the species hypothesis that is best supported at the moment (Malicky 2005, Neu 2018). For better readability, we will hence refer to the two genetically differentiated species found in Central Germany throughout the article as *S. flavicorne* and *S. personatum*.

As a first step, loci present in at least 50% of all individuals were tested for fixed single nucleotide polymorphisms (SNPs), i.e., diagnostic markers, between the two *Sericostoma* species. The respective scaffolds were extracted from the draft genome. For 217 loci, the sequences were checked for the presence of a palindromic restriction enzyme recognition site at the fixed, diagnostic SNPs. Three restriction enzyme motifs occurring frequently in the analyzed subsets were EcoRV (GATATC), NdeI (CATATG), and PvuII (CAGCTG), respectively. We used in-house python scripts (available on request) to check for the presence of diagnostic sites in the remaining loci with fixed SNPs. Subsequently, primers for amplification of regions containing the selected SNPs were designed with Geneious 6.0.6 (Biomatters Ltd). To enable the later multiplexing of different markers, the primers were chosen to result in (i) different fragment lengths of the undigested fragments and (ii) different fragment lengths of the digested fragments for markers cut by the same restriction enzyme.

In a final step, the primer sequences were checked for the potential amplification of multiple fragments. Hence, they were mapped against the *Sericostoma* genome (GenBank accession NCQO00000000.1) using the blastn megablast algorithm (Altschul et al. 1990) and primer pairs with multiple hits were excluded from the test set.

### Samples

To test the reliability and performance of the potential markers, 80 *Sericostoma* sp. specimens from 17 locations from two different regions in Germany were investigated. Additionally, markers were tested for four *Oecismus monedula* specimens. A detailed list of locations is given in Table 1. Specimens collected in 2013 and 2014 were those studied by Weigand et al. (2017). All specimens were morphologically determined at least to genus level, while samples of 2013, 2014 and 2017 were determined by SD and HW to species level using the diagnostic criteria outlined in Weigand et al. (2017). Samples were stored in 96% Ethanol prior to extraction.

**Table 1.** Overview of sampling sites, number of analyzed specimens per site, DNA extraction method used (SaPr = salt precipitation and/or CH = Chelex), and morphological identification level.

**Site**	**Stream**	**Coordinates [N/E**]	**Geographical region**	**Year**	**Number of samples**	**Extraction method**	**Identification level**
Svb	Silvertbach	51.644583 7.230139	North Rhine-Westphalia	2017	7	SaPr	species
D	Diemel	51.420806 8.808667	North Rhine-Westphalia	2017	7	SaPr	species
V	Volme	51.241611 7.531167	North Rhine-Westphalia	2017	2	SaPr	species
1OW	Tributary Lüderbach	50.475897 9.295219	Hesse	2018	5 + 1 *O. monedula*	CH	genus
2KS	Tributary Rammholzer Wasser	50.331768 9.619321	Hesse	2018	5 + 3 *O. monedula*	all samples with CH, 2 *O. monedula* also with SaPr	genus
3OE	Tributary Lohrbach	50.116317 9.462612	Hesse	2018	7	all samples with CH, 2 *Sericostoma* also with SaPr	genus
2OS	Schmale Sinn	50.33576 9.696705	Hesse	2018	6	all samples with CH, 2 *Sericostoma* also with SaPr	genus
Han	Hannebecke	51.30635 8.41007	North Rhine-Westphalia	2014	1	SaPr	species
Val	Valme	51.31553 8.40354	North Rhine-Westphalia	2014	2	SaPr	species
Nie	Nier	51.31380 8.35892	North Rhine-Westphalia	2014	4	SaPr	species
Bra	Brabecke	51.29762 8.40043	North Rhine-Westphalia	2014	3	SaPr	species
Nes	Nesselbach	51.17494 8.41878	North Rhine-Westphalia	2014	3	SaPr	species
Roe	Röhr	51.27247 8.05820	North Rhine-Westphalia	2014	7 + 1 hybrid	SaPr	species
Sch	Schwarze Ahe	51.20499 7.72184	North Rhine-Westphalia	2014	3	SaPr	species
Kru	Krummenau	51.07383 7.71277	North Rhine-Westphalia	2014	9 + 2 hybrids	SaPr	species
Sor	Sorpe	51.19883 8.42851	North Rhine-Westphalia	2013	3	SaPr	species
Sil	Silberbach	51.03180 8.05244	North Rhine-Westphalia	2014	3	SaPr	species

### DNA extraction and genus assignment

DNA was extracted using two different protocols: For samples collected in 2018, tissue was taken from legs and thoracic muscle and DNA extracted following a Chelex-based extraction protocol, by incubating tissue samples in 150 µl 10% (w/v) Chelex 100 (Bio-Rad) at 95 °C for 15 minutes in total, vortexing the samples every 5 minutes. For samples from 2013, 2014, and 2017, tissue was taken from the abdomen of specimens. To avoid contamination, gut content was removed before extraction. DNA was extracted according to a salt precipitation protocol (Weiss and Leese 2016) and DNA was eluted in TE minimum or deionized water and stored at 4 °C. For comparison of extraction methods, six samples of 2018 were additionally extracted with the salt precipitation protocol (see Table 1). Assignment of specimens to *S. personatum*/*flavicorne*, or respectively *O. monedula*, was verified by CO1 barcoding. The CO1 gene fragment was amplified via polymerase chain reaction (PCR) using forward primer LCO_mod (5’- TTC TAC AAA TCA TAA AGA TAT TGG AAC -3’) and reverse primer FL_rueck1 (5’- TAA GCTCGG GTA TCA ACG TCT AT -3’; Leese 2004, modified after Folmer et al. 1994). PCR for each sample was performed in a 25 µl reaction volume with 1× PCR buffer, 0.2 mM dNTPs, 0.5 μM of each primer, 0.008 U/μl VWR Taq DNA-Polymerase (VWR), and 1 μl of DNA template. Standard thermal cycling conditions were used for CO1 amplification (initial denaturation at 94 °C for 2 min; 38 cycles of 20 s at 94 °C, 30 s at 46 °C, 60 s at 72 °C, and final extension at 72 °C for 5 min). The obtained PCR products were purified as described in Weiss and Leese (2016) and bidirectionally sequenced at GATC-Biotech AG (Cologne, Germany).

### RFLP marker PCR

For RFLP marker validation, a PCR amplification was carried out for all developed primer pairs in separate reactions for 39 samples. Primers are listed in Table 2. Each PCR amplification was conducted in 25 µl reaction volumes with the QIAGEN Multiplex PCR Plus Kit, using 1x MasterMix, 0.5 µM of each primer, and 1 µl of template DNA. Thermal cycler settings were identical for all nine primer pairs (initial denaturation 5 min at 95 °C; 40 cycles of 30 s at 95 °C, 90 s at 50 °C, 90 s at 72 °C, and final extension for 10 min at 65 °C). Amplification success was verified on 1% TBE agarose gels. Subsequently, primers were also tested in multiplex reactions for 58 samples to facilitate the process and make species identification as fast and cheap as possible. When multiplexing, primer pairs EcoRV1 and EcoRV4, EcoRV2 and EcoRV5, NdeI1 and NdeI4, as well as NdeI5 and NdeI8 were combined to avoid overlapping of bands with equal sizes in the agarose gel. The PCR protocol remained the same, primer concentration was 0.5 µM for all primers individually, and the amount of deionized water was reduced accordingly. For the enzyme PvuII only one RFLP primer pair was chosen, hence, no multiplexing was possible.

**Table 2.** List of primers with corresponding restriction enzymes and expected fragment sizes for *S. flavicorne* (Sf) and *S. personatum* (Sp).

**Enzyme**	**Primer name**	**Primer sequence (5‘-3‘)**	**T_m_ [°C**]	**Species with restriction site**	**Fragment size *Sf* [bp**]	**Fragment size *Sp* [bp**]
**EcoRV**	EcoRV1_fw	GTGCTTCTGTCCTGTTATTC	54.0	Sp	397	229
	EcoRV1_re	TTCAAACTTGCAAAAATGCC	54.1			168
	EcoRV2_fw	AAAGAGGCGATTAACTTTCG	54.0	Sp	542	165
	EcoRV2_re	CACATTATGAACACCACACA	53.8			377
	EcoRV4_fw	AATCACTAAAACTGCCAACC	54.1	Sp	739	217
	EcoRV4_re	CTTGTACCCGTTATCGAGAG	55.1			522
	EcoRV5_fw	GAGTTCTGATCCTGTTTGTG	54.0	Sp	470	129
	EcoRV5_re	TGGCCTAGCTCAATAAATGA	54.1			341
**NdeI**	NdeI1_fw	TCTTCTGGTTCTAGGGAAAA	54.1	Sp	667	354
	NdeI1_re	ACGAAGACTGAACTCTCAAT	53.2			313
	NdeI4_fw	TCAGCATGACAGGTGAATAT	54.1	Sf	302	440
	NdeI4_re	ACAAAATGAGGCAAGTGAAT	54.0		138	
	NdeI5_fw	TGTTTGATGGATTCCTCAGA	54.0	Sf	379	547
	NdeI5_re	TGCCTCTCATCCTATTGATC	54.0		168	
	NdeI8_fw	TTATTCGCGCCATACTTTAC	53.9	Sf	455	761
	NdeI8_re	ATGGTCTTACCCGTTTAGAG	54.2		306	
**PvuII**	PvuII2_fw	GCATAACCGACAATGTGTAA	54.0	Sf	564	876
	PvuII2_re	CTAGCTCATTTCCTTTGTGG	54.0		312	

### Enzymatic digestion and species determination

All PCR products were digested with the respective enzymes in 30 µl reaction volumes as follows: 10 µl of unpurified PCR product, 2 µl of Green Buffer (to directly load digested PCR products on agarose gels), 1 µl of either FastDigest Eco321 (isoschizomer or EcoRV), FastDigest NdeI or FastDigest PvuII (all Thermo Scientific), and 17 µl deionized water. Incubation was conducted at 37 °C for 5 min for Eco321 and PvuII, and for 60 min for NdeI. Digested PCR products were visualized on 2% TBE agarose gels and compared to a 100 to 1000 bp ladder to determine the size of the fragments. Species were identified by comparing the resulting patterns to the expected fragment lengths (Table 2).

## Results

Amplification success differed slightly between candidate markers. Generally, the success rate was high for all tested single and multiplex reactions (Suppl. material 1, Table S1) with primer pairs NdeI5 and PvuII2 showing greatest success of 98.9% (n = 92) across all samples analyzed and NdeI8 showing lowest success rate of 69.2% (n = 94). NdeI8 worked much better in single reactions (89.7%; n = 29) than in multiplex reactions (60.0%; n = 65), which explains its overall lower success rate. The extraction method (Chelex or salt precipitation) did not noticeably influence the success rate. However, visualization of restricted fragments from Chelex extractions resulted in weaker bands than from salt-extracted samples, but the bands were distinctive with both methods (Suppl. material 2, Fig. S1). Further, no evidence for amplification bias between the different sampling regions was found.

**Figure 1.**
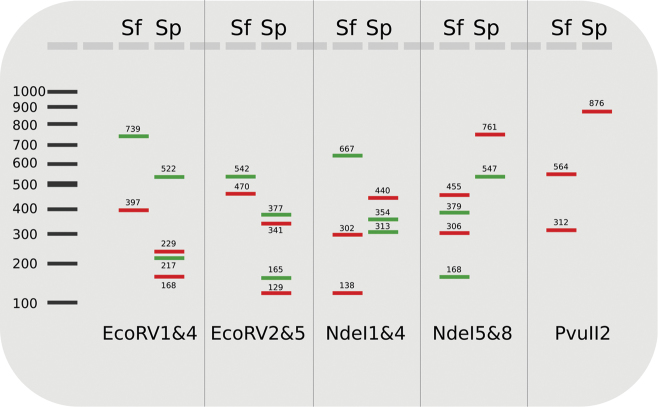
Schematic overview of the different multiplex and single RFLP fingerprints for *S. flavicorne* (Sf) and *S. personatum* (Sp). Fragment lengths are given above/below bands. Red: EcoRV1, EcoRV5, NdeI4, NdeI8, and PvuII2; Green: EcoRV4, EcoRV2, NdeI1, NdeI5.

A schematic overview of the band patterns predicted after the primer design for both species is shown in Fig. 1. This pattern was supported by laboratory tests. In the following, fragment patterns for the different multiplexed markers plus the single marker PvuII2 are described for both species, and the corresponding fragment lengths are given in Fig. 1. For EcoRV1&4 *S. flavicorne* had uncut fragments for both markers, while four cut fragments were visible for *S. personatum*. A similar pattern was generated with EcoRV2&5. NdeI5&8 generated an inverted pattern, with two intact amplicons for *S. personatum* and four cut fragments for *S. flavicorne*. An intermediate picture was obtained by NdeI1&4, where in *S. flavicorne* NdeI1 remained uncut, while NdeI4 was cut in two fragments. In contrast, for *S. personatum* NdeI4 remained uncut and NdeI1 was cut respectively. Lastly, PvuII2 did not cut in *S. personatum*, but in *S. flavicorne*. For restrictions with FastDigest NdeI, incomplete digestion was observed sometimes, meaning that bands of the original length were still slightly visible on the agarose gel in addition to the expected smaller cut fragments. For three specimens originating from three different streams (Nier, Brabecke and Sorpe), the EcoRV5 primer pair produced a fragment that was about 200 bp shorter than the expected 470 bp.

When the PCR amplification was successful, enzymatic restriction and species assignment based on the resulting fragment patterns was successful for all *S. flavicorne/personatum* individuals, i.e., all 38 individuals (excluding hybrids) previously identified to species level by ddRAD seq (Weigand et al. 2017) could be unequivocally identified. For each of the 16 individuals identified morphologically (2017 samples) using the criteria by Weigand et al. (2017), the RFLP assignment patterns confirmed the morphological identification.

For the four tested *O. monedula* specimens, PCR was successful only for four primer pairs (EcoRV1, EcoRV2, EcoRV4 and PvuII2): For the PvuII2 primer pair a product of ~550 bp was visible when amplified from two of the samples additionally extracted by salt precipitation, while no product was visible when amplified from Chelex extraction (4 individuals). EcoRV1 yielded a fragment of ~900 bp in single marker assessment that was not visible in multiplex tests. In general, bands on the agarose gels were faint in single marker assessment and barely visible in multiplex approaches. EcoRV2 yielded the same pattern after restriction as expected for *S. flavicorne* while EcoRV4 created the same pattern as specimens of *S. personatum*.

The three tested hybrids amplified for all markers but created intermediate results after restriction (Suppl. material 2, Fig. S2). Two hybrids (H2 and H3) were F1 hybrids and the third specimen (H1) was a backcross individual with *S. personatum* according to Weigand et al. (2017). For EcoRV1&2, NdeI1&4 and NdeI5&8 markers, restriction yielded multiband patterns not indicating either of the species, but uncut and cut fragments were present in the same samples, indicating heterozygosity in the hybrid samples. The combination of EcoRV2&5 and PvuII2 indicate the pattern for *S. flavicorne* for H2, but again create multiband patterns for the other two samples. For PvuII2 the pattern created also an additional band at ~950 bp for H1 and H3, which is not expected for any of the species. As expected from the ddRAD data, the inconclusive patterns created here, indicated that the specimens were neither *S. personatum* nor *S. flavicorne*, but rather a hybrid between both.

## Discussion

In this study, we developed and tested nine RFLP markers to distinguish the two sibling caddisfly species *S. flavicorne* and *S. personatum* in Germany, which cannot be identified using COI barcoding. An advantage of the RFLP approach is that no sequencing is required and thus species assignment is possible directly after PCR and a short restriction incubation, followed by simple agarose gel visualization. Since all primer pairs amplify with the same PCR settings, all markers can be tested in simultaneous reactions, with the possibility of multiplexing two primer pairs each.

Different factors can impact on the identification success of the RFLP markers. First, good amplification success for the correct amplicons in the PCR is needed to enable subsequent restriction digestion. We found high success rates of ≥ 90% for all single reactions and ≥ 80% for all multiplex reactions, with the exception of NdeI8 (only 60%). For three individuals, an alternative, shorter fragment was amplified for EcoRV5, which might be caused by a local sequence variant. This phenomenon may increase in frequency when extending the geographic range (ascertainment bias). Second, the restriction enzymes need to digest the PCR fragments reliably. While this was the case for FastDigest Eco321 (Isoschizomer of EcoRV) and FastDigest PvuII, incomplete digestion was sometimes observed for FastDigest NdeI, even though incubation time was set to 60 min as advised by the manufacturer. Finally, the DNA extraction method should not influence the reliability of the results in order to provide a robust method for application in different laboratories. The two extraction methods tested here, i.e., the salt precipitation and the Chelex approach, did not systematically impact the amplification or restriction success. Still, DNA extracted with salt precipitation showed stronger bands on the agarose gels after PCR and after restriction than Chelex extracted samples, indicating a lower yield of PCR products for Chelex extractions. Despite the lower output, the fragments were still successfully amplified. Hence, for a quick and inexpensive *S. personatum*/*flavicorne* identification, Chelex extraction is well-suited, since it is simpler, cheaper and especially much faster than most other extraction methods.

Besides the different technical aspects, we also tested if the RFLP method works for the target species across a broader geographic scale than used in the study of Weigand et al. (2017) (2013/2014 samples – max. distance: 55 km). Therefore, further samples from North Rhine-Westphalia were included (2013/2014 & 2017 samples – max. distance: 112 km), as well as samples from a second region in Germany, Hesse (min. distance to North Rhine-Westphalian samples: 99 km). Between the two regions and within North Rhine-Westphalia, no bias was detected in species identification. This indicates that the markers cannot only be used for species identification for the region in which the markers were developed (2013/2014 samples), but also for specimens from a broader geographical scale. However, we highly recommend to further test the markers with individuals previously identified morphologically or ideally identified using ddRAD seq, when analyzing specimens from other regions or countries.

As the study of Weigand et al. (2017) detected rare hybrids between the two *Sericostoma* species, we evaluated the possibility of misidentifying them as one of the parental species with the RFLP-markers. For the two F1 generation hybrids and the backcross with *S. personatum*, most of the markers showed a heterozygote pattern (meaning cut and uncut fragments per marker), which enables their clear distinction from the parental species. While these results are promising for hybrid detection, the sample size here was small (n=3) and thus results have to be interpreted cautiously.

In addition to *S. flavicorne*/*personatum*, we also tested our RFLP-markers with *O. monedula* samples. These species can co-occur in the streams and can be difficult to distinguish morphologically. In contrast to *S. personatum*/*flavicorne*, amplification success was low for *O. monedula*. No fragments were amplified for several markers, but with EcoRV2 the *S. flavicorne* and with EcoRV4 the *S. personatum* amplicon was generated. Furthermore, two markers (EcoRV1 and PvuII2) generated additional fragments for *O. monedula*, when the DNA was extracted via salt precipitation. These in general weak bands, were almost absent when using DNA from the Chelex extraction or when multiplexing markers. Hence, scoring only individuals successfully amplified for several markers with unambiguous species identification as well as excluding specimens with bands expected only for *O. monedula*, allows to clearly distinguish the two *Sericostoma* species from *O. monedula*. Individuals not fulfilling these criteria cannot be directly assigned to *O. monedula*, especially if only few markers amplify successfully. While the specific PCR products found for *O. monedula* may allow an unambiguous species identification of this species with our markers, our sample size of *O. monedula* is too low for any validation. We currently recommend the use of COI barcoding to clearly assign them to *O. monedula* as for this purpose DNA barcoding works reliably. It should also be noted that the proposed approach does not work for community-based DNA assessments (DNA metabarcoding) but only for individual specimen-based approaches and thus would inquire an additional analysis step.

In summary, the markers introduced in this study are an easy-to-use, cheap, and reliable alternative to CO1 barcoding for determining the problematic sister species *S. personatum* and *S. flavicorne*. They were applied with high amplification and restriction success rates per marker in single and multiplex approaches. The latter allows to halve material costs and reaction times (Zangenberg et al. 1999). By using several markers, reliable results can be obtained even if one primer pair fails to amplify as each marker creates clear and easily distinguishable patterns on its own. Additionally, the probable hybrids between the two *Sericostoma* species can only be clearly identified when using several markers. If for economic reasons only a subset of the here presented markers should be applied, we recommend to select the EcoRV RFLP markers and PvuII2, as they enable the identification of hybrids and the exclusion of *O. monedula* specimens in the dataset evaluated here.

It is important to note that the specimens tested herein only come from a small part of the total species range; therefore, the proven success of identification with the markers is limited to the regions tested. Supposedly, the method will give informative results in different areas as well, which remains to be established in the future.

## Supplementary materials

Supplementary material 1 Supplementary Table S1 Data type: molecular data Explanation note: Overall amplification success (%) of primer pairs, multiplex, and single reaction approaches combined. https://binary.pensoft.net/file/327111This dataset is made available under the Open Database License (http://opendatacommons.org/licenses/odbl/1.0/). The Open Database License (ODbL) is a license agreement intended to allow users to freely share, modify, and use this Dataset while maintaining this same freedom for others, provided that the original source and author(s) are credited.Sonja Darschnik, Florian Leese, Martina Weiss, Hannah Weigand

Supplementary material 2 Supplementary figures Data type: molecular data Explanation note: Figure S1 – three hybrid samples (H1, H2, H3) after restriction for all markers. Figure S2 – comparison of six samples (two of each *S. personatum*, *S. flavicorne* and *O. monedula*) with assessed with PvuII2 after restriction, salt extraction protocol on the left and Chelex extraction on the right. https://binary.pensoft.net/file/327112This dataset is made available under the Open Database License (http://opendatacommons.org/licenses/odbl/1.0/). The Open Database License (ODbL) is a license agreement intended to allow users to freely share, modify, and use this Dataset while maintaining this same freedom for others, provided that the original source and author(s) are credited.Sonja Darschnik, Florian Leese, Martina Weiss, Hannah Weigand

## References

[B1] Altschul SF Gish W Miller W Myers EW Lipman DJ (1990) Basic Local Alignment Search Tool. Journal of Molecular Biology 215(3): 403–410. 10.1016/S0022-2836(05)80360-2 2231712

[B2] Botosaneanu L (2001) *Sericostoma flavicorne* Schneider,1845 and *S. schneideri* Kolenati, 1848: two distinct species and the correct use of their names (Trich., Sericostomatidae).- Bulletin de la Société entomologique de France 106(5): 518–520.

[B3] Folmer O Black M Hoeh W Lutz R Vrijenhoek R (1994) DNA primers for amplification of mitochondrial Cytochrome c Oxidase Subunit I from diverse metazoan invertebrates. Molecular Marine Biology and Biotechnology 3(5): 294–299. 7881515

[B4] Hagen HA (1859) Die Phryganiden Pictet’s. Nach Typen bearbeitet. Entomologische Zeitung des Entomologischen Verein zu Stettin 20: 131–170.

[B5] Hebert PDN Cywinska A Ball SL deWaard JD (2003) Biological identifications through DNA Barcodes. Proceedings of the Royal Society B: Biological Sciences 270(1512): 313–321. 10.1098/rspb.2002.2218 PMC1691236 12614582

[B6] Kirby W Spence W (1826) An introduction to entomology: Or Elements of the Natural History of Insects: With Plates. Longman, Rees, Orme, Brown, and Green, London,1818–26.

[B7] Leese F Wagner R (2005) The “*Sericostoma*-Problem” – Molecular genetic, chemotaxonomic, and autecological approaches (Trichoptera: Sericostomatidae). Lauterbornia 54: 161–63.

[B8] Leese F. (2004) Molecular genetic, chemotaxonomic, and autecological investigations of European Sericostomatidae (Insecta: Trichoptera). PhD Thesis, Ruhr-Universität Bochum, Bochum.

[B9] Malicky H (2005) Ein kommentiertes Verzeichnis der Köcherfliegen (Trichoptera) Europas und des Mediterrangebietes. Linzer biologische Beiträge 37(1): 533–596.

[B10] Morales HE Pavlova A Joseph L Sunnucks P (2015) Positive and purifying selection in mitochondrial genomes of a bird with mito-nuclear discordance. Molecular Ecology 24(11): 2820–2837. 10.1111/mec.13203 25876460

[B11] Neu PJ (2018) Trichoptera-RP – die Köcherfliegenseiten von P. J. Neu. Informationen zu Ökologie, Taxonomie und Verbreitung der Köcherfliegen in Deutschland. http://www.trichoptera-rp.de [accessed 02.02.2019]

[B12] Peterson BK Weber JN Kay EH Fisher HS Hoekstra HE (2012) Double Digest RADseq: An inexpensive method for de novo SNP discovery and genotyping in model and non-model species. PLoS ONE, 7(5). 10.1371/journal.pone.0037135 PMC3365034 22675423

[B13] Pitsch T (1993) Zur Larvaltaxonomie, Faunistik und Ökologie mitteleuropäischer Fließwasser-Köcherfliegen (Insecta: Trichoptera). Schriftenreihe des Fachbereichs Landschaftsentwicklung Sonderheft S8: 322. 10.14279/depositonce-4809

[B14] Schlick-Steiner BC Steiner FM Seifert B Stauffer C Christian E Crozier RH (2010) Integrative taxonomy: A multisource approach to exploring biodiversity. Annual Review of Entomology 55(1): 421–438. 10.1146/annurev-ento-112408-085432 19737081

[B15] Schmidtke R Brandt S (1995) Ökologische und chemotaxonomische Untersuchungen zur Arttrennung von *Sericostoma flavicorne* Schneider 1845 und *Sericostoma personatum* (Spence in Kirby & Spence 1826) (Trichoptera: Sericostomatidae). Lauterbornia 22: 69–83.

[B16] Schneider WG (1845) Verzeichnis der von Prof. Loew im Sommer 1842 in der Türkei und Klein-Asien gesammelten Neuropteren nebst kurzer Beschreibung der neuen Arten. Stettiner Entomologische Zeitung 6: 153–155.

[B17] Sipahiler F (2000) Redescription of *Sericostoma flavicorne* SCHNEIDER, 1845 and a new species of genus *Sericostoma* LATREILLE from Turkey (Trichoptera, Sericostomatidae). BRAUERIA (Lunz am See, Austria) 27: 23–25.

[B18] Toews DPL Brelsford A (2012) The biogeography of mitochondrial and nuclear discordance in animals: biogeography of mito-nuclear discordance. Molecular Ecology 21(16): 3907–3930. 10.1111/j.1365-294X.2012.05664.x 22738314

[B19] Toews DPL Mandic M Richards JG Irwin DE (2014) Migration, mitochondria, and the yellow-rumped warbler. Evolution 68(1): 241–255. 10.1111/evo.12260 24102562

[B20] Weigand H Weiss M Cai H Li Y Yu L Zhang C Leese F (2017) Deciphering the origin of mito-nuclear discordance in two sibling caddisfly species. Molecular Ecology 26(20): 5705–5715. 10.1111/mec.14292 28792677

[B21] Weiss M Leese F (2016) Widely distributed and regionally isolated! Drivers of genetic structure in *Gammarus fossarum* in a human-impacted landscape. BMC Evolutionary Biology 16(1). 10.1186/s12862-016-0723-z PMC4966747 27473498

[B22] Weiss M Weigand H Weigand AM Leese F (2018) Genome-wide single-nucleotide polymorphism data reveal cryptic species within cryptic freshwater snail species – the case of the *Ancylus fluviatilis* species complex. Ecology and Evolution 8(2): 1063–1072. 10.1002/ece3.3706 29375779 PMC5773296

[B23] Zangenberg G Saiki RK Reynolds R (1999) Multiplex PCR. PCR Applications, 73–94. Elsevier. 10.1016/B978-012372185-3/50007-9

