# Supplementary material for: When barcoding fails: development of diagnostic nuclear markers for the sibling caddisfly species Sericostoma personatum (Spence in Kirby & Spence, 1826) and Sericostoma flavicorne Schneider, 1845
Source: Zookeys. 2019 Aug 20;872:57–68. doi: 10.3897/zookeys.872.34278 (PMC6711935; doi:10.3897/zookeys.872.34278)
Supplement: Supplementary material 1 [file zookeys-872-057-s002.docx]

| **Primer pair** | **single reactions** | **successful amplification** | **amplification success [%]** | **multiplex reaction** | **successful amplification** | **amplification success [%]** | **total reactions** | **successful amplification** | **amplification success [%]** |
| --- | --- | --- | --- | --- | --- | --- | --- | --- | --- |
| EcoRV1 | 35 | 34 | 97.41 | 65 | 58 | 89.23 | 100 | 92 | 92 |
| EcoRV2 | 27 | 27 | 100 | 65 | 62 | 95.38 | 92 | 89 | 96.74 |
| EcoRV4 | 27 | 26 | 96.30 | 65 | 62 | 95.38 | 92 | 88 | 95.65 |
| EcoRV5 | 41 | 39 | 95.12 | 65 | 53 | 81.54 | 106 | 92 | 86.79 |
| NdeI1 | 35 | 24 | 97.14 | 65 | 64 | 98.46 | 100 | 98 | 98 |
| NdeI4 | 27 | 26 | 96.30 | 65 | 63 | 96.92 | 92 | 89 | 96.74 |
| NdeI5 | 27 | 26 | 96.30 | 65 | 65 | 100 | 92 | 92 | 98.91 |
| NdeI8 | 29 | 26 | 89.66 | 65 | 39 | 60 | 94 | 65 | 69.15 |
| PvuII2 | 92 | 91 | 98.91 | - | - | - | 92 | 91 | 98.91 |
